# Sporadic Burkitt lymphomas of children and adolescents in Chinese: a clinicopathological study of 43 cases

**DOI:** 10.1186/1746-1596-7-72

**Published:** 2012-06-22

**Authors:** Cheng-Feng Bi, Yuan Tang, Wen-Yan Zhang, Sha Zhao, Xiao-Qing Wang, Qun-Pei Yang, Gan-Di Li, Wei-ping Liu

**Affiliations:** 1 Department of Pathology, West China Hospital of Sichuan University, Guoxue street 37, Chengdu, Sichuan, 610041, China

## Abstract

**Background:**

To investigate the clinical and pathologic features as well as the *MYC* translocations of childhood Burkitt lymphoma (BL) from China.

**Methods:**

Fourty-three cases of childhood BL were retrospectively investigated in morphology, immunophenotype, genotype, treatments and survival analysis.

**Results:**

Clinically, there was a marked male predominance in sex distribution (M: F = 9.75:1); abdomen was the most frequent extranodal sites of involvement (46.5 %), followed by jaws and facial bones (16.3 %). Two third of the patients were in stageI ~ II. Morphologically, 69.76 % of the cases showed classical histologic features, while 30.24 % of them showed greater nuclear pleomorphism in size and shape. Five cases (11.6 %) were positive for EBER1/2. Thirty-one of the 40 cases (77.5 %) had the aberration of *IGH/MYC* translocation while 7 (17.5 %) had non-*IGH/MYC* translocation. Thirty patients (69.7 %) received operation and/or chemotherapy while 13 patients (30.3 %) received no treatment. Twenty-seven patients (62.8 %) died of the tumor, 16 alive, with the average survival time 4.9 and 48.7 months respectively. High IPI, advanced clinical stage, increased serum level of LDH and no chemotherapy received as well as tumor size ≥10 cm were related to the lower survival rates of the tumor.

**Conclusions:**

Several differences were showed in this group of BL, including a much higher ratio of male patients, more cases in stageII, clinically inconsistent treatment and a very poor outcome.

**Virtual slides:**

The virtual slide(s) for this article can be found here http://www.diagnosticpathology.diagnomx.eu/vs/1552295877710135

## Background

Burkitt lymphoma (BL) is defined by the updated WHO classification (2008) as a B-cell lymphoma with an extremely short doubling time that often presents in extranodal sites or as an acute leukemia [1]. BL is a highly aggressive non-Hodgkin lymphoma (NHL) and characterized by *C-MYC* translocation. Three clinical variants of BL are recognized, including endemic BL, sporadic BL and immunodeficiency-associated BL. Sporadic BL is seen throughout the world, mainly in children and adolescents. Histologically, BL is characterized by diffuse infiltration of monomorphic medium-sized neoplastic cells with basophilic cytoplasm and numerous mitotic figures; some cases may have tumor cells with greater pleomorphism in shape and nuclear size, which was designated as “atypical BL” or “Burkitt like lymphoma” [2]. Although the incidence of BL is low, accounting for only 1 ~ 2 % of all lymphomas in western countries, it is one of the most common types of malignant tumors in children and young adults [1]. In the past decades, there were many reports of sporadic BL, including cases from Asian countries and areas [3,4], However, only a few cases of Chinese BL has been reported in the English literature. In this study, 43 cases of BL of children and adolescents from southwest China were retrospectively investigated for the clinical and pathological features.

## Patients and methods

### Patient selection

Sixty cases of initially diagnosed Burkitt lymphoma of children and adolescents (age ≤18 years) were received from the Department of Pathology, West-China Hospital of Sichuan University between 1990 and 2006. The specimens fixed in 10 % neutral formalin (pH 7.2), were processed by routine methods and embedded in paraffin. All cases were reviewed by two expert heamatopathologists individually. The criteria used to select the cases were as follows: (1) cases diagnosed as Burkitt lymphoma (BL) with a typical growth pattern and a high proliferation index (PI) as established by Ki-67 staining (in general, >90 % nuclei positivity), occasionally with a some degree of pleomorphism in nuclear size and shape, in accordance with WHO classification (2008) [1]; (2) The neoplastic cells expressed B-cell differentiation antigens (CD20), germinal center markers of CD10 and /or BCL-6 but not BCL-2. 17 of 60 cases were excluded because of insufficient materials for further studies. Thus, total 43 cases were included in current study. The clinical history of each patient was reviewed for age at diagnosis, gender, number and location of the tumor, staging, performance status, treatment regimens, complete blood count (CBC), bone marrow aspiration/biopsy, serum level of lactate dehydrogenase (LDH) and international prognostic index (IPI). The staging was according to the system proposed by Murphy and Hustu and modified by Magrath [5]. The protocol of this study was approved by the Institutional Review Board or ethical committee of West China Hospital of Sichuan University.

### Immunohistochemistry

Immunohistochemistry was performed on paraffin embedded tissue sections using the Envision method (Dako, Gene Ltd, China). Antigen retrieval techniques were applied as needed for each specific antibody. The following antibodies were used: CD20, CD45RO, CD10, BCL-6 (Zymed, Zhongshan, China); CD3ϵ, BCL-2, C-myc and TdT and Ki-67 (Neumarkers, Maixin, China). DAB was used as a substrate and the positive signal was dark brown in color. The Ki-67 stain was assigned a percentage value that was calculated by positive nuclei staining per 1000 tumor cell nuclei in each case.

### In situ Hybridization for Epstein-Barr virus (EBV) encoded RNA

In situ hybridization (ISH) was carried out with a fluorescence-labeled oligonucleotide probe complementary to two Epstein-Barr virus-encoded small RNAs, EBER-1 and EBER-2 (EBER1/2, Dako, Denmark, No.Y520001). Rabbit anti-FITC antibody conjugated with alkaline phosphatase (Dako, Denmark) was used to combine with the probe, whereas NBT/BCIP was used as a substrate. The dark blue- purple hybridizing signal was located in cell nucleus. The tumor was considered positive for EBV encoded RNAs if more than 20 % of the tumor cells showing reactivity.

### Fluorescence In situ Hybridization for IGH/MYC and non-IGH/MYC

Locus-specific interphase (LSI) fluorescence in situ hybridization (FISH) was performed on paraffin-embedded tissue sections. LSI MYC tri-color probe for the *t(8;14)* (Vysis, Gene Ltd, China) were used to detect IGH/MYC; LSI MYC dual color break apart rearrangement probe was used to detect split of *MYC* on chromosome 8 (Vysis, Gene Ltd, China). Lymphomas with *MYC* breakpoint without fusion of MYC to IGH locus were considered “other *MYC* translocations”. Specifically, deparaffinized sections were pretreated by pepsin digestion (30 min) in distilled water and subsequently incubated in pepsin solution (100ug/ml, 10–30 min) at 37^0^ C to increase DNA accessibility. Sections were then fixed in 10 % neutral formalin for 5 min, dehydrated through increasing ethanol series and air-dried. 5ul of probe mix was applied to the tissue section and covered with a 10-mm cover slip. Both probe and target DNA were simultaneously denatured at 80^0^ C for 10 min and incubated for up to 42 ~ 48 hours. Hybridization signals were analyzed under the fluorescent microscope (BX51, OLYMPUS) with appropriate filters. The images were captured using Applied imaging system. A total of 200 nuclei were scored, and any abnormal pattern which was present in excess of the cutoff limits (>10 % of cells) was considered significant. *RAJI* cell line was used as a positive control.

### DNA extraction and polymerase chain reaction (PCR) for the *t*(14;18)

Genomic DNA from paraffin-embedded tissue samples was extracted by phenol-chloroform procedures. Successful DNA extraction was confirmed by amplification of 110 bp fragment of β-globin. The primers of BIOMED-2 system were used for the *t*(14;18)(BCL2/IGH) analysis. The experiment was according to the procedure of van Dongen et al. [6]. The PCR products were electrophoresed in 10 % nondenaturing polyacrylamide gel (acrylamide:biacrylamide ratio of 29:1), and silver staining was applied in visualization of the results.

### Follow up and statistical analysis

Clinical follow-up data were available for all of the forty-three patients. Lymphoma-specific survival time was calculated by determining the time from the date of diagnosis to the date of death or last follow-up, and was analyzed using the Kaplan-Meier method. For comparison of the various parameters, Fisher exact test was applied. A *p* value of 0.05 was considered statistically significant. All statistical analysis was performed using SPSS software for Windows, version 13.0.

## Results

### Clinical manifestations

**Figure 1:**
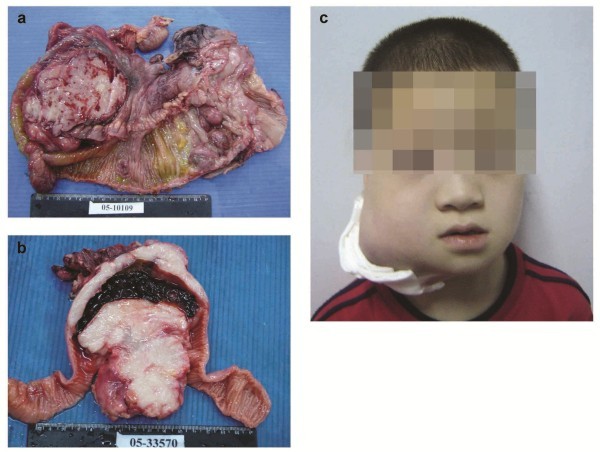
**The macroscopic photograph of Burkitt lymphoma. (a)** A 12-years boy presented with abdominal lesions. There was a huge mass within the mesentery and multiple polyploid lesions were present in the cecum; **(b)** a 6-years boy had a jejunum mass, with thickening of intestinal wall and hemorrhage; **(c)** an 8-years boy presented with a big facial bone mass.

From 1990 to 2006, children and adolescents BL accounted for 0.3 % (60/18960) of all malignant lymphomas, 0.8 % (60/7429) of all non-Hodgin lymphoma and 7.1 % (60/850) of all lymphomas of children and adolescents (age ≤18 years) in our hospital. The clinical features of the patients were listed in Additional file 1: Table S1 and summarized in Table 1. There were 39 boys and 4 girls with male to female ratio 9.75:1. The Ages at diagnosis ranged from 2 to 18 years with the mean and median age of 10.3 and 9 years, respectively. 36 patients (83.7 %) presented with extranodal lesions while seven patients (16.3 %) displayed cervical or mandibular lymphadenopathy at presentation. Twenty cases (20/43, 46.5 %) presented with abdominal masses, followed by head and neck involvement in 14 cases (14/43, 32.6 %). The mesentery with or without greater omentum involvement represented the most frequent extranodal site of involvement , accounting for 20.9 % of the cases, followed by ileocecum (8 cases, 18.6 %). Jaw and facial bone mass were presented in 7 cases (16.3 %) (Figure 1). Constitutive B symptoms such as fever, night sweat and weight loss were recorded in 17 patients (39.5 %). Clinical presentation varied according to the anatomic sites of involvement, e.g. in patients with abdominal masses, abdominal pain, distension, diarrhea as well as intestinal obstruction were the main complains or signs; in patients with jaw and cervical lymph node involvement, rapidly enlarged masses were the common findings; whereas in patients with nasopharyngeal or tonsilar masses, the symptoms included nasal obstruction, epistaxis and dysphagia. Except for one patient with Burkitt Leukemia, no hepatosplenomegaly was recorded in the other 42 patients. Comprehensive clinical examination data were collected in 34 patients, with 24 patients presented with low (low and low-intermediate) IPI scores and 10 patients with high (high and high-intermediate) IPI scores respectively. Anemia, elevated white blood cell count and thrombocythemia, and increased serum level of LDH were found in 52.9 %, 35.3 %, 61.8 % and 67.9 % of the patients respectively. According to the scheme proposed by Murphy and Hustu and modified by Magrath, 28 patients (66.7 %) were in stage I and II; nine (21.4 %) were in stage III, including two of IIIA and one of IIIB, and the remaining five (11.9 %) were in stage IV, including four patients with central nervous system lesions and one patients with bone marrow involvement. One patient suffered from Burkitt leukemia, in whom hepatosplenomegaly, increased lymphocyte count as well as bone marrow involvement were presented at initial presentation.

**Table 1:** Clinical features (n = 43)

**Contents**	**Patients**	
	**NO.**	**%**
**Sex**		
Male	39	90.7
Female	4	9.3
**Age (years)**		
Range	2-18 10.3 9	
Mean		
Median		
**Sites**		
abdomen	20	46.5
Jaws and facial bones	7	16.3
superficial lymph node	7	16.3
tonsils	3	7.0
nasopharynx	3	7.0
inner canthus	1	2.3
cerebellum	1	2.3
peripheral blood & bone marrow	1	2.3
**B symptom**	17	39.5
**Bulky disease**		
<10 cm	35	81.4
≥10 cm	7	16.3
**Lab findings (n = 34)**		
anemia	18	52.9
Increased WBC count	12	35.3
Increased PLT number	21	61.8
Increased serum level of LDH	23	67.6
**Staging (n = 43, Jude and Murphy)**		
I / II	8/20	19.1/47.6
III / IV	9/6	21.4/11.9
**IPI (n = 34)**		
Low (0–2)	24	70.6
High (3–4)	10	29.4
**Therapy (n = 30)**		
Surgery alone	10	33.3
Chemotherapy alone	11	36.7
Surgery and chemotherapy	9	30
**Follow up (n = 43, months)**		
Dead	27	62.8 (average 4.9 m)
Alive	16	37.2 (average 48.7 m)

### Pathological findings

**Figure 2:**
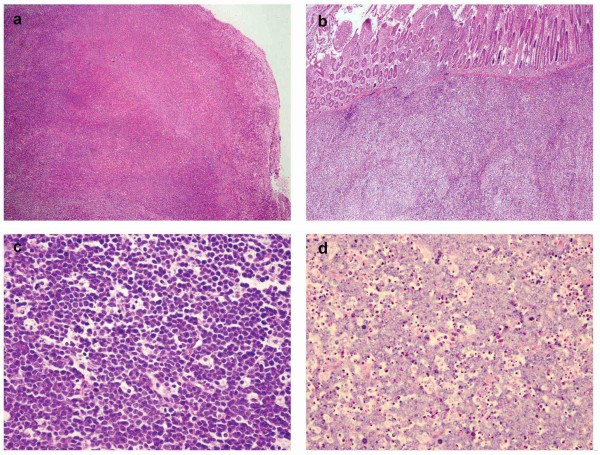
**Histological feature of Burkitt lymphoma. (a)** A nodal lesion showed the architecture of lymph node was totally effaced (H&Ex40); **(b)** intestinal lesion showed tumor cells were diffusely infiltrated in the submucosa and also intruded into mucosa. (H&Ex40); **(c)** “starry sky” and “square off” pattern were obviously seen. (H&Ex400); **(d)** a nodal lesion displayed a more pleomorphic feature with prominent nucleoli resembling “immunoblast”. Abundant apoptosis and mitoses are present (H&Ex400)

The size of the tumors ranged from 1.6x1.5x1.0 cm to 20.0x20.0x15.0 cm, in which 69.0 % of the cases were larger than 5 cm in diameter. Various degrees of coagulative necrosis and hemorrhage were presented in most of the specimens. The morphologic features of the cases are summarized in Table 2. Classical histologic features of Burkitt lymphoma was presented in 30 of 43 cases (69.8 %), which showed medium-sized neoplastic cells arranging in sheets with remarkably monomorphic consistence in size and shape. The nuclei were round or oval with finely clumped and dispersed chromatin and 2 ~ 4 basophilic medium sized, centrally situated nucleoli. The cytoplasm was scarce to moderate and basophilic. Some tumor cells presenting with dusty cytoplasm and invisible nucleoli resembled lymphoblasts. In the remaining 13 cases (30.2 %), the neoplastic cells were less monomorphic but showed greater nuclear pleomorphism in size and shape, some of the tumor cells displayed more prominent nucleoli resembling “immunoblast”. In addition, small number of tumor giant cells was also seen in 3 cases. “Starry Sky” growth pattern and “squared off” feature were observed in 36 (83.7 %) and 31 (72.1 %) of the cases, respectively. Abundant apoptosis was notable in 40 (93.0 %) of the cases, and numerous mitotic figures were detected in all of the tissue samples; Furthermore, more than 50 of mitotic figures in 10 high power field (HPF) was detected in 9 (20.9 %) of the cases, whereas 5 to 50 of mitotic figures in 10 HPF was presented in 34 (79.1 %) of the cases (Figure 2). No epithelioid granuloma was found in current group of the cases, and “blood cell lake” was seen in 2 (4.7 %) cases.

**Table 2:** Morphologic features (n = 43)

**Morphology**	**NO.**	**%**
**“Starry sky” pattern**	36	83.7
**Squared off feature**	31	72.1
**Coagulative necrosis**	7	16.3
**Greater nuclear pleomorphism**	15	34.9
**Tumor giant cells**	3	7.0
**Abundant apoptosis**	40	93.0
**Mitotic figures**		
>50/10HP	9	20.9
5-50/HP	34	79.1
**Epithelioid granulomas**	0	
**Blood cell Lake**	2	4.7

For the seven cases with lymph node involvement, the architecture of the node was completely effaced by diffuse infiltration of neoplastic lymphoid cells. The other morphologic features were similar as those described above.

### Immunophenotype and EBV status

**Figure 3:**
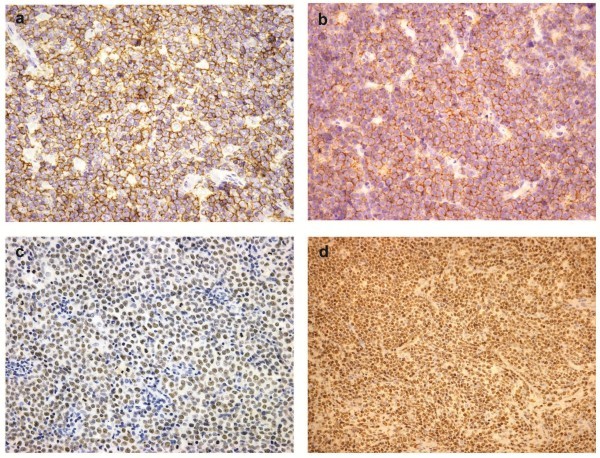
**Immunohistochemistry of Burkitt lymphoma.** Tumor cells were positive for CD20 **(a)**, CD10 **(b)**, BCL-6 **(c)**; nearly 100 % of tumor cells were positive for Ki-67 **(d)** (x200)

**Figure 4:**
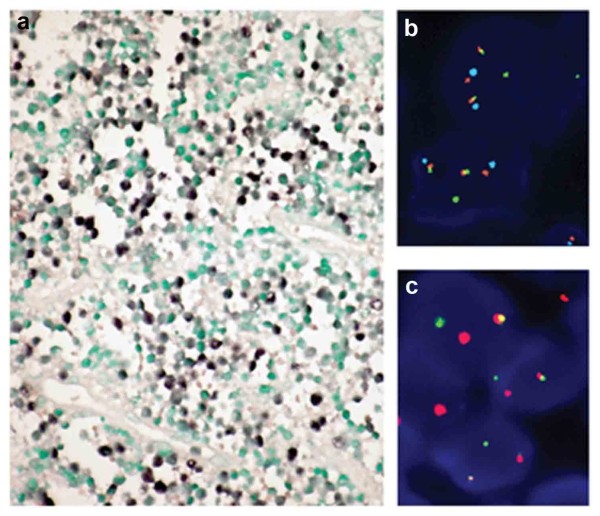
(a) EBV-ISH showed positive for EBER1/2 (x400); (b) FISH study using tri-color dual fusion probe showed two fusion signals indicating translocation between MYC and IGH (the aqua signal were CEP 8); (c) break-apart probe detected a break of MYC, which indicating other MYC translocation in the absence of MYC/IGH translocation.

The results of immunohistochemistry and EBER-ISH were summarized in Table 3. The neoplastic cells of all cases expressed CD20 as well as CD10. In addition, 40 of 43 cases (93.0 %) were also positive for BCL-6 and 4 cases (9.3 %) were positive for CD45RO. No case showed a positive reaction for BCL-2, TdT or CD3ϵ. The neoplastic cells expressed C-MYC protein in 33 of the 43 cases. Ki-67 index was greater than 90 % in all of the cases and greater than 95 % in 37 cases (Figure 3). Five cases (11.6 %) were positive for EBER1/2 with the proportion of positive cells ranging from 20 to 60 % (mean 40 %) (Figure 4a).

**Table 3:** Results of IHC, EBER-ISH, PCR and FISH

**Markers**	**+/Number**	**%**
**IHC (n = 43)**		
**CD20**	43/43	100
CD3ϵ*	0/43	0
**CD45RO**	4/43	9.3
**CD10**	43/43	100
**BCL-6**	40/43	93.0
**BCL-2**	0/43	0
**TDT**	0/43	0
**C-MYC**	33/43	76.7
**Ki-67**		
≤95 %	6/43	14.0
>95 %	37/43	86.0
**EBER-ISH (n = 43)**	5/43	11.6
**BCL-2/IGH by PCR (n = 39)**	0/39	0
**FISH (n = 40)**		
* IGH/MYC*	31/40	77.5
Other *MYC* translocation	7/40	17.5

* Polyclonal.

### MYC gene translocation and BCL-2/IGH gene rearrangement analysis

The FISH study was successfully performed in 40 cases, the results are also summarized in Table 3. Of all 38 cases showing signal split with *C-MYC* break-apart FISH probes, 31 (77.5 %) were positive for *IGH/MYC* fusion FISH signals, indicating the *t*(8;14)(q24;q32) translocation; 7 (17.5 %) were negative for *IGH/MYC*, suggesting *IGL/MYC* fusion or other rare translocation with *MYC*(Figures 4b and 4c); in two cases the tumors were negative for both probes*. The t*(14;18) (BCL-2/IGH) *t*(14;18) analysis by DNA amplification using PCR method was performed in 39 cases and no BCL-2/IGH translocation was identified.

### Treatment, Follow-up and Survival Analysis

Clinical treatment information were collected for all of the 43 patients. Nineteen patients (23.3 %) received surgical resection, nine with combined chemotherapy subsequently. Eleven patients (25.6 %) received chemotherapy alone, whereas, unfortunately, the remaining 13 patients (30.4 %) received no further treatment after the diagnosis was established by biopsy. Among 20 patients who received chemotherapy, ten patients received CHOP-like regimen as initial therapy, while the remaining ten patients received intensive regimen including HYPER-CVAD or LMB. Among 20 patients who received chemotherapy with or without surgical resection, complete remissions (CR) and partial remissions (PR) were achieved in 11 and 4 patients respectively, whereas the remaining 5 patients failed to show any response to chemotherapy.

**Figure 5:**
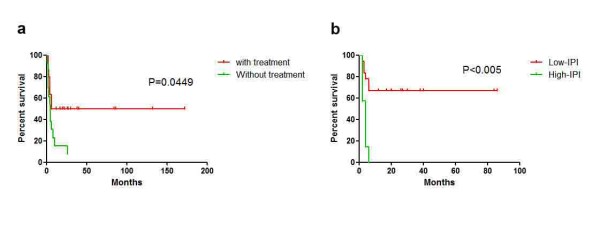
(a) The overall survival curve of 30 patients with treatment and 13 patients without treatment; (b) In treated patients, high IPI (n = 9) had worse prognoses than those with low IPI (n = 16) (p < 0.001).

**Figure 6:**
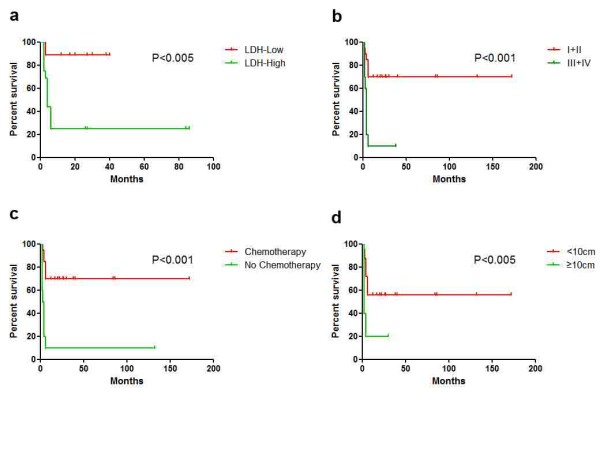
Survival curves of 30 treated patients with regard to different parameters showed (a) Patients with normal level of LDH(n = 11) had better prognoses over those with high level of LDH (n = 23)(P < 0.005); (b) patients with low stages (I & II, n = 20) had better prognoses over those with advanced stages (III & IV, n = 10)(P < 0.001); (c) patients who received chemotherapy (n = 20) had favorable outcomes than those who did not receive chemotherapy (n = 10)(P < 0.001); (d) patients with tumor size ≥ 10 cm (n = 5) has worse outcomes than those with size < 10 cm (n = 25)(P < 0.005).

Follow-up data was available for all of the 43 patients (100 %). The mean follow-up time was 86.5 months, ranging from 1 to 172 months. Twenty-seven of them (62.8 %) died of the tumor with average survival of 4.9 months. At the end of follow-up, 16 patients were alive with average survival of 48.7 months. Patients without any treatment were all died soon after diagnosis, whereas patients with either surgical resection or chemotherapy treatment had better outcomes (Figure 5a). For the 30 patients received treatment, lymphoma specific overall survival (OS) were analyzed in regard to various parameters, including IPI, age (≤14 and > 14 years), staging (I/II and III/IV), elevation of WBC and thrombocythemia, increased serum level of LDH, with or without chemotherapy, size of the tumor (<10 cm and ≥10 cm) and histomorphology (with or without greater nuclear pleomorphism). As a result, high IPI, advanced clinical stages (III and IV), without chemotherapy received, bulky disease (≥10 cm) and increased serum level of LDH were correlated to the poorer survival (Figures 5b and 6). Although there was no prognostic difference between patients treated with CHOP-like regimen and intensive regimen according to survival analysis, eight of ten cases (80 %) with intensive regimen achieved CR after treatment, whereas only three cases (30 %) in CHOP-like group achieved CR.

## Discussion

Herein, we reported clinicopathological features of 43 cases of pediatric BL from Southwest China. Pediatric BL accounts for 7.1 % of all childhood lymphomas at our hospital. It showed classical pathological features of BL with typical morphology, immunohistochemical findings, cytogenetic changes, and clinical features. However, our study showed that a relatively lower incidence of BL in a representative pediatric population in Southwest China, marked male predominance, and particularly poorer survival, partially due to insufficient therapy.

BL accounts for about 30 ~ 50 % of childhood lymphomas in the Western countries [1]. The reason for lower incidence in our cohort is currently unknown. Cairo et al. [7] reviewed 470 patients from USA, the male to female ratio was 4:1 in that cohort. Chuang et al. [3] studied 17 pediatric BL from Taiwan, the male to female ratio was 1.8:1; whereas in current study, all patients but four were boys, the ratio of male to female was 9.75:1. The gender ratio of newborns in this part of China was about 1.2:1, and there was no gender bias in patient selection. Our findings may suggest BL were more prone to affect boy in this area. Further larger scale of study is required to confirm this finding.

Majority of sporadic BL present with abdominal masses, whereas jaw is rarely affected; majority of BL patients (70 %) present at advanced stages [1].Whereas in our study, close to one-thirds of patients presented with head and neck lesions, together with the patients with superficial lymph node involvement, resulting in a higher percentage of cases in stage I/II in our cohort.

BL is a neoplasm of mature B-cell lineage, which was thought to arise from germinal center B-cells [8]. Therefore, the tumor cells often express germinal center associated markers, such as CD10 and Bcl-6. Ki-67 index are always nearly 100 % [9]. In current study, expression of CD10 and BCL-6 were 100 % and 93 %, respectively, and Ki-67 index was all beyond 90 %. No BCL-2 expression or BCL-2/IGH gene rearrangement was detected in all examined cases. The results are similar to that reported in the literatures. The MYC translocation is a hallmark of Burkitt lymphoma. The most common type of *MYC* translocation is the *t*(8;14)(q24;q32), accounting for 80 % of BL; In the remaining 20 %, one of two variants is identified: *t*(2;8)(p12;q24) or *t*(8;22)(q24;q11) [9]. In this study, *MYC* translocation was found in 95 % of the cases, including 77.5 % of *IGH/MYC and* 17.5 % of other translocations. Although *MYC* translocation detection by FISH was thought to be the “gold criterion”, 9 % ~ 14 % of the cases may lack a detectable *MYC* translocation by FISH, the explanation for which is uncertain [10-12].

The frequency of EBV infection in sporadic BL varied largely according to geographical distributions, e.g. 0 % to 8 % in Japan; 25 % of reported Taiwanese cases; 34 % of Israel; 47 % of Argentina; 52.6 % of Brazil; 80 % of India [13-16]. Some researchers suggested that general conditions of health and social economic status may play a role. In our study, 11.6 % of the cases were positive for EBER1/2 by in situ hybridization, and it is lower than that of average level (20 % ~ 40 %) of EBV infection reported [17]. Although EBV could be detected in almost all the endemic BL, to date, its significance in pathogenesis of the sporadic BL is still controversy. In addition, Bellan [18] studied the cell origin of BL by semi-nested PCR to amplify the VDJ rearrangement of IG heavy chain (*V*_*H*_) genes and sequencing analysis, the results suggested that EBV-positive and EBV-negative BL may originate from two distinct subsets of B-cells, pointing to a particular role for the germinal center reaction in the pathogenesis of the tumor. Thorley’s research showed the similar results as well [19].

Although BL was less common in adults and the present study mainly focused on children and adolescents, pediatric and adult sporadic BLs shared similar phenotypic and genotypic features [8]. A study based on gene expression profiling by Klapper et al. [20] showed the same results, that no differences were detectable between pediatric and adult molecular BL with regard to gene expression and chromosomal imbalances.

The following morphologic features, such as sheets of monomorphic and medium-sized neoplastic cells, “starry sky” growth pattern, numerous mitotic figures as well as apoptotic bodies favor the diagnosis of BL. Molecular profiling studies also showed that the histomorphological diagnosis of classical Burkitt lymphoma is quite robust [11,21]. Furthermore, in combination with immunohistochemistry, *MYC* translocation by FISH, diagnosis of BL is usually easy to make in most instances. However, the neoplastic cells of BL may also display a variety degree of pleomorphism in nuclear size and shape, which was described as Burkitt like lymphoma (BLL) and categorized as a variant of BL in WHO Classifications of 2001 [2]. The major problem is to distinguish BL with greater pleomorphism from DLBCL. Gene-expression profiling has confirmed that BL is a homogenous molecular phenotype clearly distinct from other B-NHL including DLBCL [8,11,21,22]. Nevertheless, it is currently impractical for us to use gene-expression profiling analysis in routine diagnosis. Some tumors with histomorphological and immunohistochemical features intermediate between BL and DLBCL, so called “gray zone lymphoma” are present and further studies are wanted for understanding the pathogenesis of this group of tumors as well as their effective therapy.

Despite the fact that BL is a highly aggressive lymphoma with a double time of 25.6 h, it is potentially curable. Intensive combination chemotherapy regimens result in cure rates up to 90 % in patients with low stage disease and 60 ~80 % in patients with advanced stage disease [1]. We reviewed the major clinical manifestations, treatment and outcome of BL reported in English literatures in the last decade (Table 4) [3,7,13,23-26]. The patients in current study had a relatively poor prognosis with both one and two years overall survival rates at only 39.5 %. The possible reasons may include, but not limited to: (1) less than 50 % of the patients received chemotherapy, and about one-third of patients gave up the further treatment; (2) the chemotherapy regimens used were inappropriate or inadequate. CHOP-like regimen, the most commonly selected therapy for DLBCL was not suitable for BL [27,28]. Short and intensive therapy, including methotrexate and Ara-C is recommended and is associated with a significant improvement in patients with BL, even for those with advanced stages [29-31]. In our study, although there was no survival difference between patients treated with CHIOP-like regimen and intensive therapy, it was striking that intensive therapy could result in CR in most patients, indicating its advantage in the initial therapy. In addition, high IPI, advanced clinical stage (III/IV), bulky disease, and increased serum level of LDH were also related to the lower survival, which suggest BL is a curable disease in children and adolescents, however, early diagnosis and therapy is crucial for improving the clinical outcome.

**Table 4:** Review and comparison of the major clinical manifestations, treatment and outcome of BL

**Authors**	**Country/Region**	**Number**	**Age (years)**	**M :F**	**Abdomen (%)**	**H & N (%)**	**LN (%)**	**CNS (%)**	**Treatment**	**Os or EFS (years)**
**Reiter A** (1995) [23]	Germany	152	0-17	3.6:1	/	/	/	4	BFM 86	EFS 79 %(1y)
										EFS 79 %(2y)
**Ertem U** (1996) [24]	Turkey	63	3-14	2:1	96.8	15.9	≤3.2 %	7.9	Ziegler’s and intensive protocol*	Os 60.0 %(1y)
										Os 57.8 %(2y)
**Cario MS** (2003) [7]	USA	470	0-21	3.7:1	/	/	/	12	COMP/LSA_2_L_2_	Os 70.0 (1y)
									COMP/D-COMP	EFS 60.0 %(1y)
									CCG-552	Os 64.5 % (2y)
									Orange/French**	EFS 58.5 %(2y)
**Boerma EG** (2004) [25]	Netherlands	66	0-15	4.5:1	>42	9	20	/	/	/
**Hassan R** (2008) [13]	Brazil	54	2-14	2:1	72	2	11	4	/	/
**Chuang SS** (2008) [3]	Taiwan	17	0-16	1.8:1	41.2	41.2	17.6	11.8	CHOP(−like)	Os 80 %(1y)
									Modified BFM regimen	Os 66.7 % (2y)
**Mbulaiteye SM** (2009) [26]	USA	296	0-14	3.7:1	21	9	56		/	/
**The current study**	China	43	0-18	9.75:1	46.5	32.6	16.3	9.3	CHOP(−like) /Hyper-CVAD/	Os 39.5 %(1y)
									LMB/ HD-MTX + Ara-c	Os 39.5 %(2y)

* Eight drug intensive protocol, generated a favorable outcome than Ziegler’s protocol.

** A protocol with Short and intensive therapy, including intensified methotrexate and Ara-C, was associated with a significant improvement in long-term EFS (80 ± 6 %).

In conclusion, we described 43 cases of childhood BL. Compared with that reported in the literature, some differences were presented in our cohort, including the higher ratio of male patients and higher percentage of patients presented with lower stages, clinically insufficient treatment and management as well as a very poor outcome of the tumor. Because BL was not common in mainland China, It is necessary for us to ameliorate the management and treatment of the tumor to improve the prognosis in the near future.

## Competing interests

There is no actual or potential conflict of interest in relation to this article.

## Authors’ contributions

Weiping Liu designed the whole study. Chengfeng Bi carried out the case collection, immunoassays, and molecular genetic studies, participated in the statistical analysis and helped to draft the manuscript. Yuan Tang and Wenyan Zhang participated in molecular genetic studies. Sha Zhao participated in case collection. Xiaoqing Wang participated in molecular genetic studies and statistical analysis. Qunpei Yang participated in the immunoassays. Gandi Li participated in design of the study and helped to confirm the diagnosis. All authors read and approved the final manuscript.

## Supplementary Material

Additional file 1 **Table S1.** Clinical manifestations and follow-up data. Click here for file
