# Supplementary material for: Sporadic Burkitt lymphomas of children and adolescents in Chinese: a clinicopathological study of 43 cases
Source: Diagn Pathol. 2012 Jun 22;7:72. doi: 10.1186/1746-1596-7-72 (PMC3414834; doi:10.1186/1746-1596-7-72)
Supplement: Additional file 1 — Table S1. Clinical manifestations and follow-up data. [file 1746-1596-7-72-S1.doc]

**Table 1 clinical features (n=43)**

| **Contents** | **Patients** | |
| --- | --- | --- |
|  | **NO.** | **%** |
| **Sex** |  |  |
| Male | 39 | 90.7 |
| Female | 4 | 9.3 |
| **Age (years)** |  |  |
| Range | 2-18  10.3  9 | |
| Mean |  |  |
| Median |  |  |
| **Sites** |  |  |
| abdomen | 20 | 46.5 |
| Jaws and facial bones | 7 | 16.3 |
| superficial lymph node | 7 | 16.3 |
| tonsils | 3 | 7.0 |
| nasopharynx | 3 | 7.0 |
| inner canthus | 1 | 2.3 |
| cerebellum | 1 | 2.3 |
| peripheral blood & bone marrow | 1 | 2.3 |
| **B symptom** | 17 | 39.5 |
| **Bulky disease** |  |  |
| ＜10 cm | 35 | 81.4 |
| ≥10 cm | 7 | 16.3 |
| **Lab findings (n=34)** |  |  |
| anemia | 18 | 52.9 |
| Increased WBC count | 12 | 35.3 |
| Increased PLT number | 21 | 61.8 |
| Increased serum level of LDH | 23 | 67.6 |
| **Staging (n=43, Jude and Murphy)** |  |  |
| Ⅰ / Ⅱ | 8/20 | 19.1/47.6 |
| Ⅲ / Ⅳ | 9/6 | 21.4/11.9 |
| **IPI (n=34)** |  |  |
| Low (0-2) | 24 | 70.6 |
| High (3-4) | 10 | 29.4 |
| **Therapy (n=30)** |  |  |
| Surgery alone | 10 | 33.3 |
| Chemotherapy alone | 11 | 36.7 |
| Surgery and chemotherapy | 9 | 30 |
| **Follow up (n=43, months)** |  |  |
| Dead | 27 | 62.8 (average 4.9m) |
| Alive | 16 | 37.2 (average 48.7m) |

**Table 2 Morphologic features (n=43)**

| **Morphology** | **NO.** | **%** |
| --- | --- | --- |
| **“Starry sky” pattern** | .36 | 83.7 |
| **Squared off feature** | 31 | 72.1 |
| **Coagulative necrosis** | 7 | 16.3 |
| **Greater nuclear pleomorphism** | 15 | 34.9 |
| **Tumor giant cells** | 3 | 7.0 |
| **Abundant apoptosis** | 40 | 93.0 |
| **Mitotic figures** |  |  |
| >50/10HP | 9 | 20.9 |
| 5-50/HP | 34 | 79.1 |
| **Epithelioid granulomas** | 0 |  |
| **Blood cell Lake** | 2 | 4.7 |

**Table 3 Results of IHC, EBER-ISH, PCR and FISH**

| **Markers** | **+/Number** | **%** |
| --- | --- | --- |
| **IHC (n=43)** | | |
| **CD20** | 43/43 | 100 |
| **CD3ε*** | 0/43 | 0 |
| **CD45RO** | 4/43 | 9.3 |
| **CD10** | 43/43 | 100 |
| **BCL-6** | 40/43 | 93.0 |
| **BCL-2** | 0/43 | 0 |
| **TDT** | 0/43 | 0 |
| **C-MYC** | 33/43 | 76.7 |
| **Ki-67** |  |  |
| ≤95% | 6/43 | 14.0 |
| >95% | 37/43 | 86.0 |
| **EBER-ISH (n=43)** | 5/43 | 11.6 |
| **BCL-2/IGH by PCR (n=39)** | 0/39 | 0 |
| **FISH (n=40)** |  |  |
| *IGH/MYC* | 31/40 | 77.5 |
| Other *MYC* translocation | 7/40 | 17.5 |

* Polyclonal

**Table 4 Review and comparison of the major clinical manifestations, treatment and outcome of BL**

| **Authors** | **Country**  **/Region** | **Number** | **Age**  **(years)** | **M :F** | **Abdomen**  **(%)** | **H & N**  **(%)** | **LN**  **(%)** | **CNS**  **(%)** | **Treatment** | **Os or EFS**  **(years)** |
| --- | --- | --- | --- | --- | --- | --- | --- | --- | --- | --- |
| **Reiter A**  (1995) | Germany | 152 | 0-17 | 3.6:1 | / | / | / | 4 | BFM 86 | EFS 79%(1y)  EFS 79%(2y) |
| **Ertem U**  (1996) | Turkey | 63 | 3-14 | 2:1 | 96.8 | 15.9 | ≤3.2% | 7.9 | Ziegler’s and  intensive protocol* | Os 60.0%(1y)  Os 57.8%(2y) |
| **Cario MS**  (2003) | USA | 470 | 0-21 | 3.7:1 | / | / | / | 12 | COMP/LSA_2_L_2_  COMP/D-COMP  CCG-552  Orange/French** | Os 70.0 (1y)  EFS 60.0%(1y)  Os 64.5% (2y)  EFS 58.5%(2y) |
| **Boerma EG**  (2004) | Netherlands | 66 | 0-15 | 4.5:1 | ＞42 | 9 | 20 | / | / | / |
| **Hassan R**  (2008) | Brazil | 54 | 2-14 | 2:1 | 72 | 2 | 11 | 4 | / | / |
| **Chuang SS**  (2008) | Taiwan | 17 | 0-16 | 1.8:1 | 41.2 | 41.2 | 17.6 | 11.8 | CHOP(-like)  Modified BFM regimen | Os 80%(1y)  Os 66.7% (2y) |
| **Mbulaiteye SM**  (2009) | USA | 296 | 0-14 | 3.7:1 | 21 | 9 | 56 |  | / | / |
| **The current study** | China | 43 | 0-18 | 9.75:1 | 46.5 | 32.6 | 16.3 | 9.3 | CHOP(-like) /Hyper-CVAD/  LMB/ HD-MTX + Ara-c | Os 39.5%(1y)  Os 39.5%(2y) |

* Eight drug intensive protocol, generated a favorable outcome than Ziegler’s protocol.

** A protocol with Short and intensive therapy, including intensified methotrexate and Ara-C, was associated with a significant improvement in long-term EFS (80 ± 6%)
